# Prognostic implications of thyroid disease in patients with atrial fibrillation

**DOI:** 10.1007/s00380-023-02341-x

**Published:** 2023-12-13

**Authors:** Anthi Vasilopoulou, Vasiliki Patsiou, Alexandra Bekiaridou, Andreas S. Papazoglou, Dimitrios V. Moysidis, Marina Spaho, Martha Zergioti, Dimitrios Kostakakis, Maria-Eirini Kyriakideli, Chrysanthi-Ioanna Lampropoulou, Anastasios Kartas, Athanasios Samaras, Amalia Baroutidou, Apostolos Tzikas, Antonios Ziakas, George Giannakoulas

**Affiliations:** 1 First Department of Cardiology, School of Medicine, Faculty of Health Sciences, AHEPA University Hospital, Aristotle University of Thessaloniki, St. Kiriakidi 1, 54636 Thessaloniki, Greece; 2 Elmezzi Graduate School of Molecular Medicine, Northwell Health, Manhasset, NY USA; 3 Feinstein Institutes for Medical Research at Northwell Health, Manhasset, NY USA; 4 Interbalkan European Medical Center, Asklipiou 10, Pylaia, Thessaloniki, Greece

**Keywords:** Atrial fibrillation, Thyroid disease, Hypothyroidism, TSH, T3

## Abstract

Atrial fibrillation (AF) is often accompanied by thyroid disease (THD). This study aimed to explore the relationship between THD and the occurrence of significant clinical outcomes in patients with AF. This post hoc analysis utilized data from the MISOAC-AF trial (NCT02941978), which enrolled hospitalized patients with AF. Patients were categorized based on their THD history into hyperthyroidism, hypothyroidism, or euthyroidism. Cox regression models were employed to calculate unadjusted and adjusted hazard ratios (aHRs). The primary outcomes of interest included all-cause mortality, cardiovascular death, and hospitalizations during the follow-up period. The study included 496 AF patients (mean age 73.09 ± 11.10 years) with available THD data, who were followed-up for a median duration of 31 months. Among them, 16 patients (3.2%) had hyperthyroidism, 141 (28.4%) had hypothyroidism, and 339 (68.4%) had no thyroid disease. Patients with hypothyroidism exhibited higher rates of hospitalization during follow-up (aHR: 1.57, 95% CI 1.12 to 2.20, *p* = 0.025) compared to the euthyroid group. Elevated levels of thyroid-stimulating hormone (TSH) were correlated with an increased risk of cardiovascular mortality (aHR: 1.03, 95% CI 1.01 to 1.05, *p* = 0.007) and hospitalizations (aHR: 1.06, 95% CI 1.01 to 1.12, *p* = 0.03). Conversely, lower levels of triiodothyronine (T3) were associated with higher risks of all-cause mortality (aHR: 0.51, 95% CI 0.31 to 0.82, *p* = 0.006) and cardiovascular mortality (aHR: 0.42, 95% CI 0.23 to 0.77, *p* = 0.005). Among patients with AF, hypothyroidism was associated with increased hospitalizations. Furthermore, elevated TSH levels and decreased T3 levels were linked to higher cardiovascular and all-cause mortality risks, respectively.

## Introduction

In the last 20 years, the prevalence of AF has increased by 33% worldwide [1]. Thyroid disease greatly contributes to an increased risk of AF occurrence [2–4] as it is known that the cardiac muscle is involved in systemic endocrine regulation [5].Therefore, current research studies investigating the connection of heart disease with endocrine disorders such as thyroid disease are searching for a potential causative pathophysiologic relation [6, 7].

In fact, the significance of serum thyroid hormone level fluctuation for AF recurrence is studied [8], while T3 levels, specifically, have been linked to myocardial ischemia recovery [9, 10]. Evidence suggests that, primarily, overt and occult hyperthyroidism are associated with AF triggering, increasing its incidence by up to twofold when compared to euthyroid patients [11–13]. Hypothyroidism, whose involvement to arrhythmias such as AF is unclear to date, appeared to be indirectly related to its occurrence through its contribution to the progression of various cardiovascular diseases [14]. Moreover, an animal-based study investigated the effects of thyroid hormone levels on arrhythmogenesis in thyroidectomized rats and showed that hypothyroidism, similarly to hyperthyroidism, may increase AF vulnerability [15].

Regarding the impact of thyroid disease (THD) coexistence in AF, studies have shown that the clinical course was partially similar in terms of stroke and bleeding risk [16, 17]. However, there are contradictory data regarding the clinical implications of hypothyroidism regarding mortality and other cardiovascular outcomes.

The aim of this retrospective post hoc analysis was to investigate the association of the THD spectrum with hard clinical outcomes in patients with AF discharged from a cardiology in-hospital unit.

## Methods

### Study design

This study constitutes a post hoc analysis of patients enrolled in the MISOAC-AF trial [18] (Motivational Interviewing to Support Oral AntiCoagulation adherence in patients with non-valvular Atrial Fibrillation, ClinicalTrials.gov identifier: NCT02941978), a prospective, two-armed, single-center, randomized controlled trial that aimed to address the effect of a motivational-educational intervention on patients’ adherence to oral anticoagulation therapy. The study was conducted in the cardiology department of AHEPA University Hospital of Thessaloniki, Greece, and a thorough analysis of the design, selection criteria, and the results have been published previously [19]. Participants were recruited in this clinical trial between December 2015 and June 2018 after providing their written, informed consent. The follow-up was completed in April 2020. The data for analysis were collected from patient–physician interviews, electronic hospital records, and the Greek National General Health Insurance System.

In this study, patients were included if they were aged over 18 years and had available data on their thyroid profile (TSH, T3, T4, or thyroid status). Based on their thyroid profile, AF patients were divided into three groups: hyperthyroidism, hypothyroidism, and euthyroidism. Further comparisons were made based solely on the serum TSH, T3, and T4 levels separately. Participants with missing data were excluded. The study complied with the ethical principles of Good Clinical Practice and the Declaration of Helsinki [20] and was approved by the Aristotle University ethics committee.

### Definition of covariates

AF was defined as previously recorded in medical history or new-onset AF occurring during hospitalization. The latter concerned irregular heart rhythm for more than 30 s, without detectable P waves, captured by a 12-lead electrocardiogram or a 24-h Holter monitor. Euthyroidism was defined as normal thyroid function with normal serum TSH (0.35–4.5 mIU/mL) and T4 (12–30 pmol/L) levels [21].

Overt hyperthyroidism was characterized by low serum concentration of TSH (< 0.35 mIU/mL) and elevated levels of T3 and/or T4 hormones. The diagnosis of overt hypothyroidism was made when serum TSH levels are abnormally high (> 4.5 mIU/mL) and levels of T4 are lower than normal [22]. T3 levels were defined as normal if they were between 0.9 and 2.8 (nmol/L).

Stroke was defined as a permanent, focal, neurological deficit confirmed by imaging modalities. Transient ischemic attack was defined as new neurologic symptoms or deficit lasting less than 24 h with no new infarction on neuroimaging (if available). Cardiovascular death (CVD) was defined as a death related to cardiac cause or stroke. All data collection was based on the standard definitions as defined in the study.

### Study outcomes

The primary outcome was all-cause mortality, and the secondary outcomes of interest were CVD and hospitalizations (any cause, AF-related and HF-related). CVD was defined as sudden cardiac death or death due to other cardiovascular causes such as acute myocardial infarction, heart failure, or stroke. AF and HF-related hospitalizations were assessed separately. Patients were followed-up until death occurred or until April 2020. The vital status of all patients was verified through the Greek Civil Registration. Other clinical events were ascertained via telephonic or in-person interviews.

### Statistical analysis

Continuous variables were summarized with means and standard deviations (SDs), while categorical variables were presented using frequencies and percentages. The baseline characteristics were compared between the three groups of the participants with either one-way analysis of variance (ANOVA) for continuous variables or Pearson Chi-square test for categorical variables as a part of a three-way cross-tabulation. Univariate Cox regression analysis was performed to assess the impact of thyroid status, T3, T4, or TSH levels on the outcomes of interest and unadjusted hazard ratios (HRs) were acquired. Variables univariately associated with the outcome of interest (i.e., gender, age in years, BMI, history of major bleeding, history of chronic kidney disease (CKD), and history of stroke) were inserted as covariates in the multivariate Cox proportional hazard models to obtain adjusted HRs (aHRs) for the investigated outcomes. The maximum number of independent variables that could be included in the final model was determined by the rule of one predictor variable for ten events of interest. Therefore, multivariate analysis for hyperthyroidism was not feasible due to the small number of events in a restricted group sample size. Survival analysis was also executed with Kaplan–Meier curves to analyze time-to-event data for patients with hypothyroidism compared to euthyroid patients, and log-rank tests were performed. The level of significance was set to a = 5% (p value < 0.05) while performing the analyses. The results are displayed with the corresponding 95% confidence intervals (CI). Data management and statistical analysis were performed using SPSS version 27 (SPSS Inc., Chicago, Illinois) software, Stata v15.1 (StataCorp, College station, Texas, United States) packages and R Statistical Software (v4.3.0; R Core Team 2023).

## Results

### Baseline characteristics and follow-up

Of the 1113 patients of the MISOAC-AF study, 496 patients (mean age 73 ± 11 years; 54% male) had available data on thyroid status and were included in this post hoc analysis. A total of 16 patients (3.2%) had a history of hyperthyroidism, 141 (28.4%) had a history of hypothyroidism, and 339 (68.4%) had no history of thyroid disease. Baseline characteristics stratified by thyroid status are displayed in Table 1. Generally, there was homogeneity among groups regarding demographic characteristics and medical history. The majority of euthyroid patients were males, while more women with AF had concomitant hypothyroidism. Persistent or permanent AF seemed to be the most prevalent types among patients with thyroid disease (16.1%), followed by paroxysmal AF or atrial flutter (13.3%).

**Table 1:** Baseline characteristics of patients with AF categorized by thyroid status

	*N*	Euthyroidism (*n* = 339)	Hypothyroidism (*n* = 141)	Hyperthyroidism (*n* = 16)	*p* value
Age, mean ± SD^a^	496	72.91 ± 11.66	73.26 ± 9.93	75.25 ± 8.91	*p* = 0.697
Gender	496				p* < 0.001
Male	268	210 (61.9%)	51 (36.1%)	7 (43.7%)	
Female	228	129 (38.0%)	90 (63.8%)	9 (56.2%)	
Smoking, *n* (%)	494	185 (54.5%)	76 (53.9%)	7 (43.7%)	*p* = 0.835
Alcohol consumption, *n* (%)	494	116 (34.2%)	41 (29.0%)	5 (31.2%)	*p* = 0.578
Coronary artery disease, *n* (%)	490	145 (42.7%)	38 (26.9%)	6 (37.5%)	***p* = 0.013**
Pulmonary disease, *n* (%)	495	61 (17.9%)	24 (17.0%)	3 (18.7%)	*p* = 0.971
Heart failure, *n* (%)	494	155 (45.7%)	75 (53.1%)	8 (50.0%)	*p* = 0.271
Chronic kidney disease, *n* (%)	495	49 (14.4%)	22 (15.6%)	1 (6.2%)	*p* = 0.594
Acute myocardial infraction, *n* (%)	495	88 (25.9%)	24 (17.0%)	2 (12.5%)	*p* = 0.068
Angina, *n* (%)	495	71 (20.9%)	20 (14.1%)	1 (6.2%)	*p* = 0.102
History of PCI^b^ or CABG^c^, *n* (%)	495				***p* = 0.005**
PCI, *n* (%)		47 (13.8%)	13 (9.2%)	5 (31.2%)	
CABG, *n* (%)		41 (12.0%)	4 (2.8%)	1 (6.2%)	
Both, *n* (%)		24 (7.0%)	11 (7.8%)	0 (0.0%)	
Congenital heart disease, *n* (%)	495	10 (2.9%)	4 (2.8%)	1 (6.2%)	*p* = 0.746
Hypertension, *n* (%)	495	270 (79.6%)	113 (80.1%)	15 (93.7%)	*p* = 0.379
Diabetes mellitus, *n* (%)	495	112 (33.0%)	44 (31.2%)	6 (37.5%)	*p* = 0.866
Vascular disease, *n* (%)	495	178 (52.5%)	64 (45.3%)	7 (43.7%)	*p* = 0.348
AF^d^- subtypes	496				*p* = 0.069
First diagnosed AF		44 (12.9%)	11 (7.8%)	0 (0.0%)	
Paroxysmal AF or atrial flutter		104 (30.6%)	59 (41.8%)	7 (43.7%)	
Persistent AF or permanent AF		191 (56.3%)	71 (50.3%)	9 (56.2%)	

*P* values that are reported in bold format were identified as statistically significant (<0.05)

P* refers to the significant difference between males and females regarding the euthyroidism and hypothyroidism categories

^a^*SD* standard deviation

^b^*PCI* percutaneous coronary intervention

^c^*CABG* coronary artery bypass graft

^d^*AF* atrial fibrillation

The median time interval between patient enrollment and the last date of follow-up was 31 months (interquartile range 10 to 52 months). During follow-up, death from any cause occurred in 200 (40.3%) patients, while the majority (72%) was due to cardiovascular causes. Among them, 134 (67%) were euthyroid patients, 62 (31%) had hypothyroidism, and 4 (2%) patients had hyperthyroidism.

### Patients with AF and comorbid hypothyroidism

**Fig. 1:**
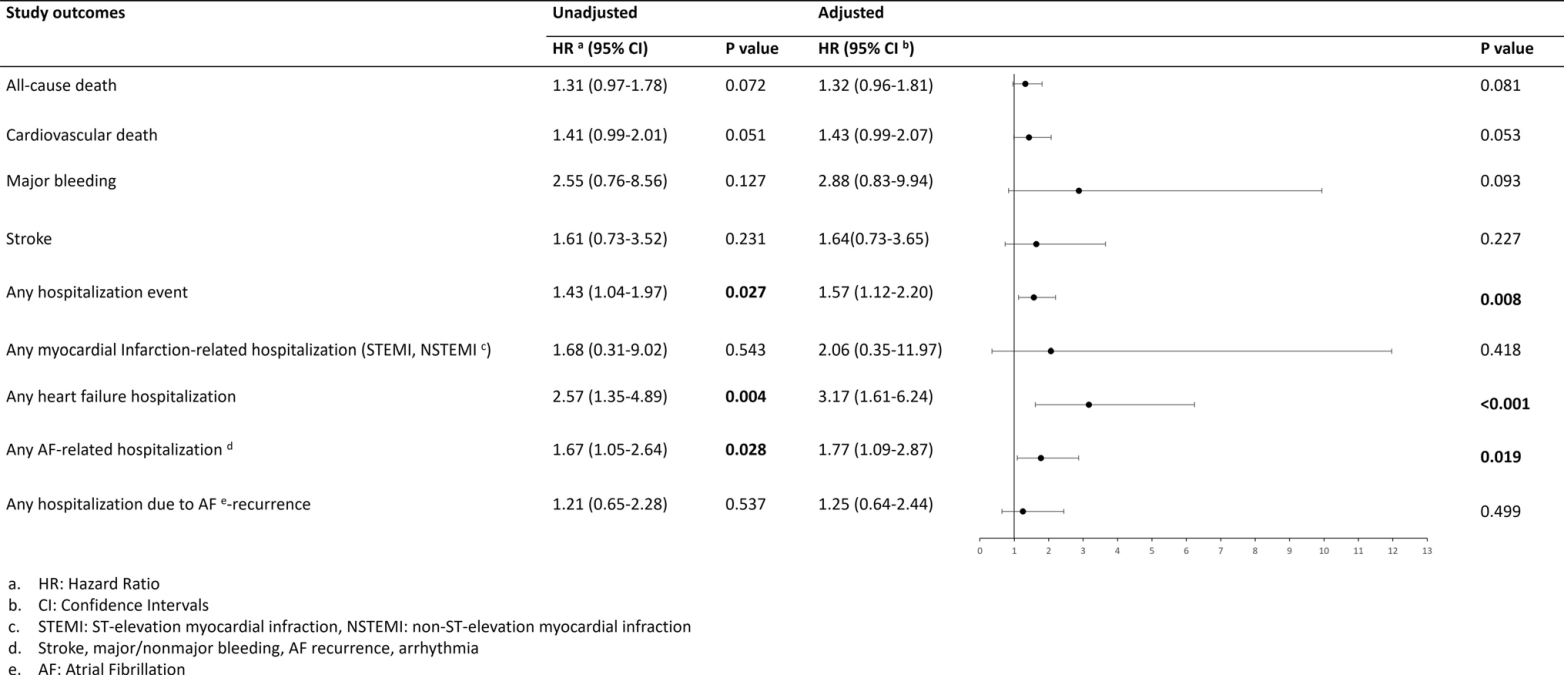
Univariate and multivariate analysis for patients with AF and comorbid hypothyroidism

**Fig. 2:**
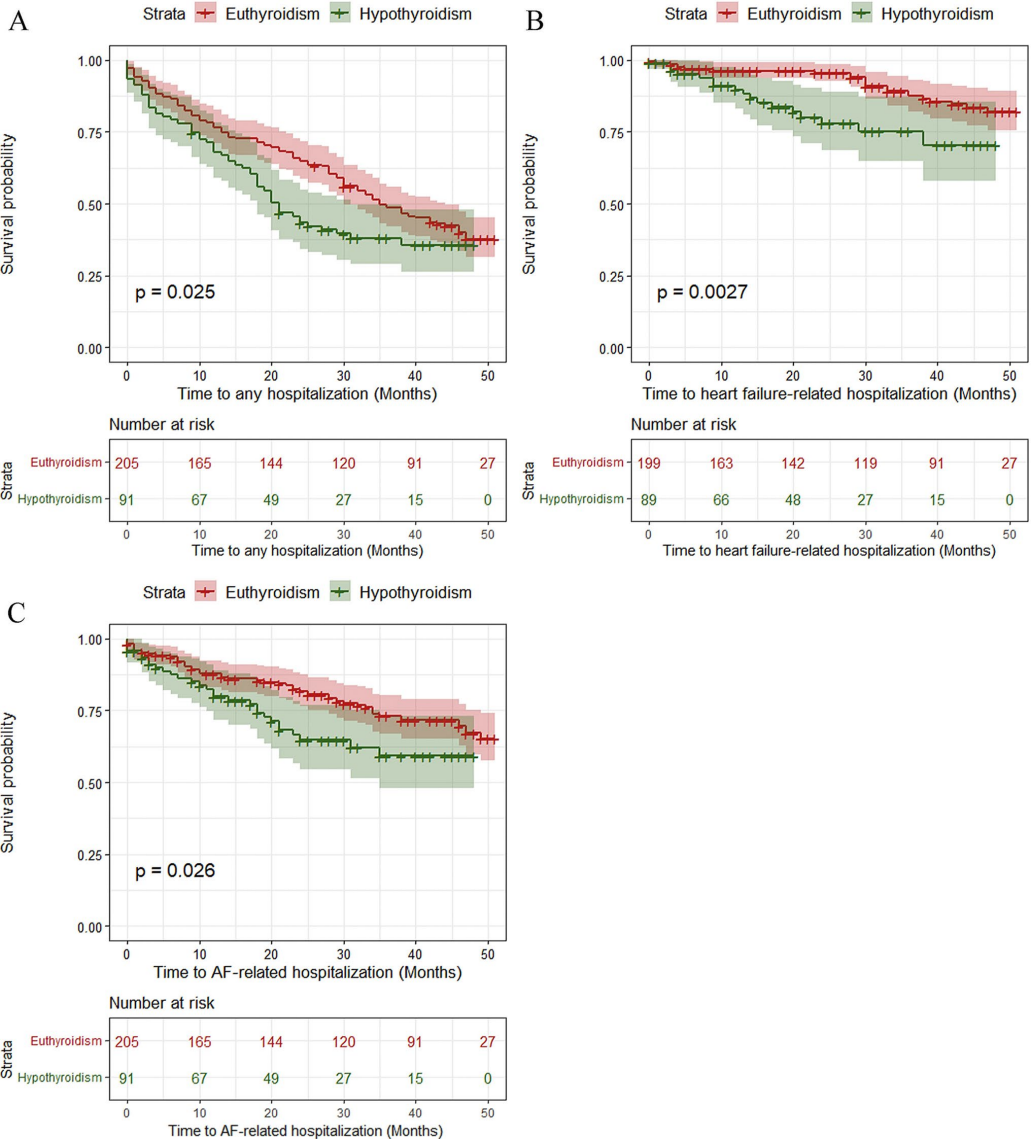
Kaplan–Meier analysis survival curves for the occurrence of **A**. any hospitalization, **B**. any heart failure-related hospitalization, **C**. any AF-related hospitalization event between AF patients with euthyroidism and hypothyroidism

Univariate and multivariate Cox regression analyses are displayed in Fig. 1. Multivariate Cox regression analysis indicated that patients with hypothyroidism had significantly higher hospitalization rates (Fig. 2A) during follow-up (aHR: 1.57, 95% CI 1.12 to 2.20, *p* = 0.025 by log-rank test) compared to those with no history of thyroid disease. More specifically, hypothyroidism was associated with an increased risk of hospitalizations due to heart failure (aHR: 3.17, 95% CI 1.61 to 6.24, *p* = 0.0027 by log-rank test, Fig. 2B) and hospitalizations related with AF complications, such as stroke, major and non-major bleeding and AF recurrence (aHR: 1.77, 95% CI 1.09–2.87, *p* = 0.026 by log-rank test, Fig. 2C). There were no statistically significant differences between the two groups of patients (hypothyroidism and euthyroidism) regarding all-cause death, CVD, and stroke.

### Patients with AF and comorbid hyperthyroidism

During follow-up, there were no statistically significant differences between patients with a history of hyperthyroidism and those with no history of thyroid disease regarding the outcomes of interest: all-cause mortality (unadjusted HR: 0.56, 95% CI 0.21 to 1.52, *p* = 0.261), risk of CVD (unadjusted HR: 0.59, 95% CI 0.18 to 1.85, *p* = 0.366), and hospital admission rates (unadjusted HR: 0.79, 95% CI 0.35 to 1.79, *p* = 0.58).

### AF patients and TSH levels

**Fig. 3:**
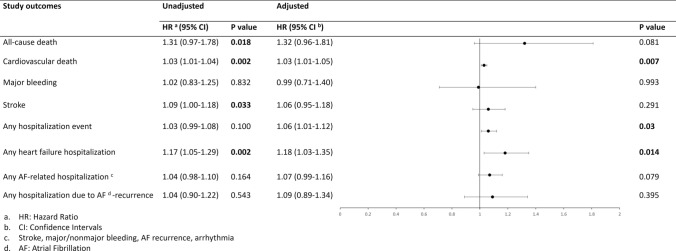
Univariate and multivariate analysis for TSH levels in patients with AF and available thyroid disease status

The HRs generated from the univariate and multivariate Cox regression analyses are displayed in Fig. 3. The multivariate analysis resulted in increased risk of CVD (aHR: 1.03, 95% CI 1.01 to 1.05, *p* = 0.007) in patients with higher TSH levels compared to those with normal or low TSH levels. High TSH levels were, also, significantly correlated with hospitalizations during follow-up (aHR: 1.06, 95% CI 1.01 to 1.12, *p* = 0.03), including those due to heart failure (aHR: 1.18, 95% CI 1.03 to 1.35, *p* = 0.014). No significant differences were noted regarding all-cause mortality (aHR:1.32, 95% CI 0.96 to 1.81, *p* = 0.081) in relation to the levels of TSH hormone.

### AF patients and T3 levels

**Fig. 4:**
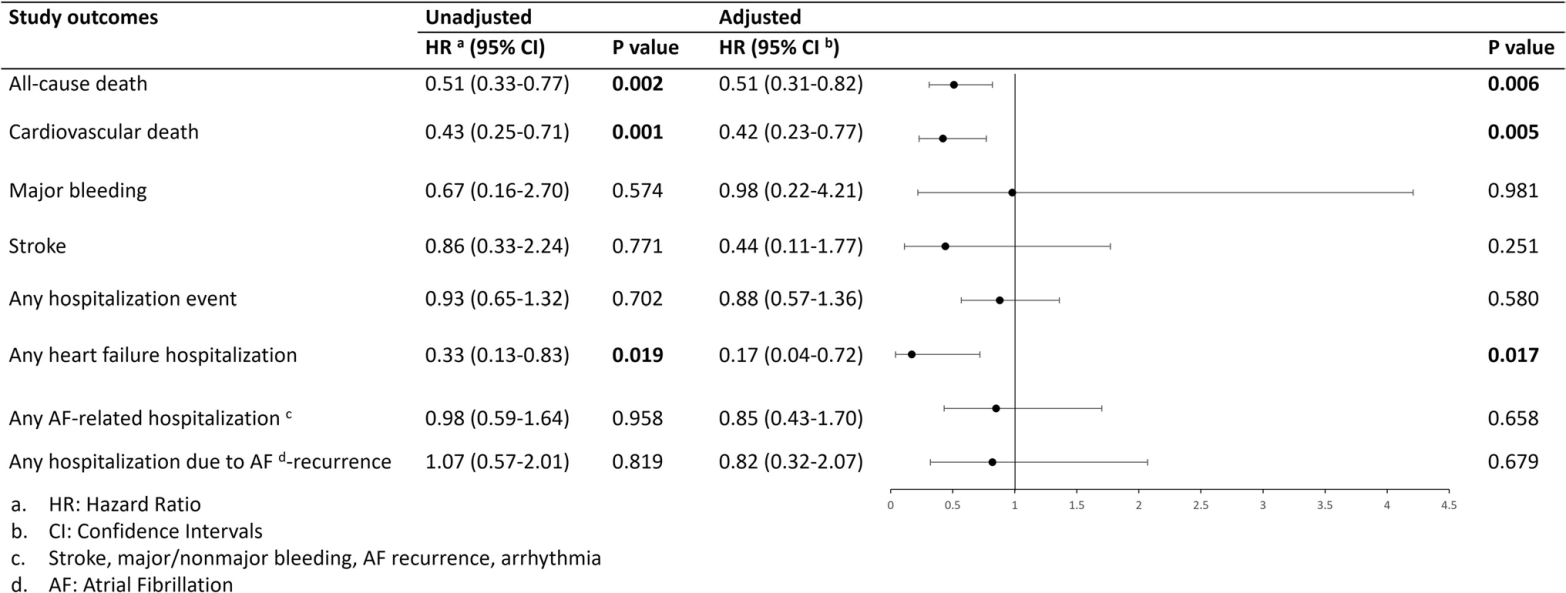
Univariate and multivariate analysis for T3 levels in patients with AF and available thyroid disease status

Lower T3 levels were correlated with higher all-cause mortality rates (aHR: 0.51, 95% CI 0.31 to 0.82, *p* = 0.006) and higher risk of CVD (aHR: 0.42, 95% CI 0.23 to 0.77, *p* = 0.005). Furthermore, lower T3 levels were associated with higher hospitalization admission rates due to heart failure (aHR: 0.17, 95% CI 0.04 to 0.72, *p* = 0.017). Results of the univariate and multivariate Cox regression analysis are depicted in Fig. 4.

### AF patients and T4 levels

Univariate Cox regression analysis showed no statistically significant relationship between T4 levels and the outcomes of interest: all-cause mortality (unadjusted HR: 0.98, 95% CI 0.92 to 1.04, *p* = 0.639), risk of CVD (unadjusted HR: 0.96, 95% CI 0.89 to 1.04, *p* = 0.411), risk of stroke (unadjusted HR: 0.84, 95% CI 0.68 to 1.04, *p* = 0.122), hospitalization due to heart failure (unadjusted HR: 1.01, 95% CI 0.93 to 1.08, *p* = 0.828), hospitalization due to AF recurrence (unadjusted HR: 1.03, 95% CI 0.99 to 1.07, *p* = 0.109), and hospitalization related to complications of AF (unadjusted HR: 1.01, 95% CI 0.97 to 1.06, *p* = 0.439).

## Discussion

In this observational post hoc analysis of the MISOAC-AF, almost one-third of hospitalized patients with AF had thyroid dysfunction. Hypothyroidism was associated with a 57% higher possibility of hospitalization compared to euthyroid AF patients, while there was a TSH level-dependent risk of CVD and HF-related hospitalization. Higher TSH levels were related to greater risk compared to normal and lower levels. Furthermore, lower T3 levels were correlated with a greater risk of all-cause death and CVD. These results add to the existing evidence regarding AF and comorbid hypothyroidism as they indicate that both hypothyroidism and isolated higher TSH levels may affect AF clinical course.

In more detail, our statistical analysis pertaining to hospitalizations indicated a threefold increase in the risk of hospitalizations due to heart failure in patients with comorbid hypothyroidism. These findings are in line with previous relevant literature [23] and further imply that hypothyroidism may contribute to heart failure progression as it can lead to a reduction in the E/A ratio and myocardial diastolic dysfunction [24]. In the Penn Heart Failure Study, a large prospective cohort of 1365 ambulatory patients with a broad spectrum of heart failure [25], thyroid dysfunction demonstrated a significant association with clinically relevant outcomes, both at baseline and longitudinally. Specifically, higher TSH and lower T3 were each associated with more severe symptoms of heart failure at baseline and increased risk of all-cause mortality. Moreover, as in other previous studies, we found that hypothyroidism was correlated with an increased incidence of AF-related hospitalizations [26, 27]. Although it has been controversial [8, 28], this finding adds to the consideration that not only hyperthyroidism, but hypothyroidism as well may predispose to more frequent AF recurrences.

Furthermore, the association of higher TSH levels with the risk of CVD highlights its possible key role to cardiovascular disease [29]. In fact, since TSH is the main biomarker for the diagnosis of subclinical hypothyroidism and there is conflicting existing data regarding its clinical course and management, the above association underscores the importance of further investigating the optimal TSH levels in conjunction with an individual’s clinical profile. This is particularly relevant when considering hormonal substitution in subclinical thyroid conditions as TSH levels may serve as an independent predictor in patients with comorbid cardiovascular diseases [30, 31].

Regarding T3 levels, it was seemed that lower T3 was correlated with all-cause and cardiovascular mortality and in patients with AF. In that context, other studies have reported that higher T3 concentration may be cardioprotective [32]. Also, the protective role of T3 hormone in heart failure course has already been discussed by existing literature [33–35]; this was evident in this study as well, since lower serum T3 levels in multimorbid AF patients were associated with a higher hospital admission rate especially due to heart failure. Nevertheless, our analysis did not yield any statistically significant relationship between T4 levels and all outcomes of interest.

Concerning hyperthyroidism, this post hoc analysis suggested no statistically significant association with the outcomes of interest. However, the relationship between hyperthyroidism and cardiovascular disease has been previously well described [36], and it is well known that hyperthyroidism contributes to the development of new-onset arrhythmias [37] and especially AF [12–40]. Admittedly, this controversy between our results and well-established literature may arise from the small number of patients with overt hyperthyroidism who participated in the study. Therefore, the sample size was not adequate to replicate the previously reported results.

Overall, although our findings should be considered in the context of our moderate sample size, they support the view that overt and subclinical hypothyroidism influence the course and recurrence of coexisting AF. Nevertheless, it would be challenging and rather vague for their prognostic significance to be predicted by a single biomarker (i.e., TSH or T3), especially in high-risk groups of patients with severe diseases at baseline [41].

### Limitations

This is a single-center, retrospective post hoc analysis of the MISOAC-AF trial. As a result, it was not designed at the outset of the trial. Hence, a considerable number of cases had to be excluded due to missing values on thyroid status, TSH, or T3, possibly resulting in selection bias. Due to its observational nature, there may be undetected biases and unmeasured or hidden confounders in the correlations of specific factors with adverse events. However, multivariable adjustments were performed for clinically relevant parameters. Our dataset consisted of older AF patients discharged from the hospital, and this may reduce the generalizability of our results to non-hospitalized patients with AF. Moreover, the proportional hazards models were formed using data on the demographics and clinical characteristics of the population as assessed at baseline. Therefore, no new comorbidities that could occur during follow-up or close to the time of the event of interest (i.e., death) were recorded. However, this single data registration at the outset of follow-up limited the retrocausality in the interpretation of our results.

## Conclusion

In this study, it was shown that patients with AF and overt hypothyroidism had increased hospitalizations while lower T3 levels and elevated TSH levels were associated with higher mortality. These findings highlight the importance of detecting and managing the thyroid hormonal status in all subjects with AF and contribute to the debate about the value of screening at-risk populations. Further real-world evidence is needed prior to reaching definite conclusions. Considering the potential for reversibility of thyroid dysfunction, this may lead to better clinical outcomes in these patients.

## Abbreviations

AF: Atrial fibrillation
(a)HR: (Adjusted) hazard ratio
CVD: Cardiovascular death
CI: Confidence intervals
HF: Heart failure
SD: Standard deviation
TSH: Thyroid stimulating hormone
